# Barriers to Promoting Advance Care Planning for Residents Living in a Sanatorium for Hansen’s Disease: A Qualitative Study of Residents and Staff in Japan

**DOI:** 10.1007/s41649-018-0059-7

**Published:** 2018-08-03

**Authors:** Mari Tsuruwaka, Rieko Yokose

**Affiliations:** 1 Division of Bioethics, Graduate School of Nursing Sciences, St. Luke’s International University, Tokyo, Japan; 2 Advanced Research Center for Human Sciences, Waseda University, Tokyo, Japan

**Keywords:** Ex-Hansen’s disease patients, Advance care planning, Sanatorium for Hansen’s disease, Staff in sanatoria, Advance directives, Japan

## Abstract

In Japan, most residents with Hansen’s disease (leprosy) live in dedicated sanatoria because of an established quarantine policy, even after being cured of the primary disease. They suffer from secondary diseases and are advancing in age, and advance care planning (ACP) is increasingly crucial for them to live their lives with dignity in a sanatorium. In this study, we have three aims: (1) to understand how to promote communication about their wishes for medical treatment, care, and recuperation; (2) to identify required assistance; and (3) to explore how to promote ACP in a sanatorium. This study is a qualitative research conducted through semi-structured interviews. The study included 57 ex-Hansen’s disease patients and 66 staff (10 doctors, 27 nurses, 23 care workers, and 6 social workers) from 10 facilities. Ex-Hansen’s disease patients were expected to consider ACP, but this was in the context of uncertainty about whether a sanatorium would close and whether there would be doctors to manage their needs. They reported being confused when staff rushed to confirm their advance directives, feeling that they were not provided with enough information before being approached. Barriers to promoting ACP were found to be insufficient of team-based care and information sharing, ex-Hansen’s disease patients’ weak interest in their end of life, and their conservative thoughts. We believe that ACP can be achieved by listening to the wishes of recovered patients through regular home care visits by nurses and everyday support by care workers. Furthermore, multidisciplinary coordination is urgently needed for promoting ACP.

## Background

### The History of Hansen’s Disease and Its Management in Japan

Hansen’s disease is a chronic infection caused by *Mycobacterium leprae*. People with Hansen’s disease are typically feared because of the prominence of sensory disturbance and deformation on visible body parts when diagnosis and treatment are delayed. Unfortunately, this led to compulsory segregation policies being introduced in Japan, with the passage of the Matter Concerning the Prevention of Leprosy Law in 1907 and the Leprosy Prevention Law in 1953. In the 1930s, the “No Leprosy Patients in Our Prefecture” movement led to quarantine of all patients with Hansen’s disease across the nation. This movement of identifying and isolating patients stoked fear of the disease and encouraged prejudice and discrimination. Meanwhile, overseas, the effectiveness of treatment by Promin was confirmed in 1943, and the global policy for Hansen’s disease shifted from quarantine to open treatment. However, in Japan, it was only with the abolishment of Leprosy Law in 1996 that national policy came in line with the international consensus.

In a sanatorium of Hansen’s disease, patients have traditionally been free to live self-sufficiently, engaged in various labors, and looked after other patients through mutual help and support. A guardianship system also existed that allowed patients with Hansen’s disease to appoint other patients to represent them after death and honor their wishes for funeral arrangements and disposition of property. Moreover, the National Leprosy Sanatoria Inpatient’s Council was set up to improve the treatment by society of patients with Hansen’s disease and to seek amendment of the Leprosy Law (Japan Law Foundation 2007).

The Leprosy Prevention Law, which had isolated patients with Hansen’s disease in a compulsory manner because of its assumed infectivity, was claimed to be unconstitutional and patients filed a lawsuit seeking damages by the government with the Kumamoto District Court in July 1998 (Ministry of Health, Labor and Welfare 2001). Despite producing a judgment in favor of the plaintiffs in May 2001, the victory did not bring about the social changes needed to ensure security among patients whose homes have already been taken away.

In 2008, the “Act to Accelerate the Resolution of Hansen’s Disease Problems” was enacted to acknowledge the economic damages, restrictions on human rights and discrimination caused by the national quarantine policy. The law obliged the government to restore the honor of patients and their families, and to guarantee complete cure and healthy living, and provide assistance with rehabilitation into society. While its objective was to create “a society where every single resident living in sanatoria after recovering from Hansen’s disease [could] live without concern,” this has not been achieved in practice. In this research, because patients are already cured, we consider them to have recovered from Hansen’s disease (or ex-Hansen’s disease patients).

### Why We Need to Focus on ACP for People Who Have Recovered from Hansen’s Disease

Interest in ACP has been growing in the medical and nursing fields (Thomas et al. 2018). It involves discussions between a patient, family members, and care providers that aim to assist with how the patient wishes to live, how to improve quality of care, and how to reduce stress for family as an illness progresses in a manner that compromises over time the autonomous decision-making capacity of the patient (Detering et al. 2010; Sampson et al. 2011). Although the definitions of ACP vary between countries (NHS 2009; Canadian Hospice Palliative Care Association 2012), and despite the lack of a global standard, a survey of 52 professionals in 4 countries emphasized that communication and not written advance directives (AD), the patients preferences for treatment, and shared decision-making in the present concerning future medical decisions were key to the process (Sudore et al. 2017). ACP typically includes documenting AD and wishes for end-of-life care (Brown et al. 1999; Molloy et al. 2000; Morrison et al. 2005). Efforts still need to be made to evaluate the quality of end-of-life care (Weathers et al. 2016). Some studies have noted the importance of self-led ACP for elderly people living in nursing homes (Molloy et al. 2000; Morrison et al. 2005). Elderly people also often place great value in communicating ACP with individuals they trust (Seymour et al. 2014), including medical caregivers (Fosse et al. 2014). Some studies about barriers to carrying out ACP obtained the same result that lack of time for healthcare providers to spend for ACP was the one commonly observed (Aultman et al. 2018; Dixon and Knapp 2018; Fulmer et al. 2018).

In Japan, as of 1 April 2018, there were approximately 1450 people with Hansen’s disease (average age 85 years) living in 14 national sanatoria for Hansen’s disease (National Institute of Infectious Disease 2018). Most of these people have been cured of the primary disease but live in the sanatoria because of the aftereffects of Hansen’s disease and their advanced ages. Ex-Hansen’s disease patients live on public welfare in which all of the costs of their medical treatment and livelihood are guaranteed by the government. Studies of Hansen’s disease have shown the different forms of stigma experienced by patients (Cross and Choudhary 2005; van Brakel et al. 2012; Kaehler et al. 2015), including the courtesy stigma experienced by family and caregivers (Dako-Gyeke 2018), especially in developing countries where the number of newly infected patients remains high. Despite full recovery and ongoing efforts to reduce stigma (Lusli et al. 2015; Peters et al. 2016), many people still perceive themselves as ill due to the permanent aftereffects on their appearance (van Harren et al. 2017).

Japan’s Ministry of Health, Labor and Welfare therefore provided a notification to 13 national sanatoria in 2014 to “establish a system to assist each inpatient’s lifestyle with respect for their wishes,” including activities of daily living (ADL), cognitive function, everyday finance management, property management, purpose in life, and wishes for terminal care (Japan’s Ministry of Health, Labor and Welfare 2014). Despite efforts to provide end-of-life care in sanatoria for ex-Hansen’s disease patients (Tsurumi 2014), no study has focused on end-of-life care and ACP among those who are now aging.

In this study, we aim to (1) understand the wishes for medical treatment, care, and recuperation among ex-Hansen’s disease patients; (2) discover the assistance needed to actualize their desired way of life; and (3) explore how to implement ACP in a sanatorium for ex-Hansen’s disease patients. This study can provide insight that could help protect and improve the dignity, humanity, and quality of life of ex-Hansen’s disease patients in Japan.

## Methods

### Study Design

A qualitative descriptive research was conducted (Sandelowski 2000), using approximately 60-min-long semi-structured interviews in Japanese. ACP was defined as “the process through which ex-Hansen’s disease patients discussed their desired lifestyle, including their wishes regarding medical treatment, care, and recuperation with their family and medical staff in advance of future decline in judgement competency.” All interviews were conducted using an interview guide after explaining this definition. The focus of interviews among ex-Hansen’s disease patients was on eliciting responses regarding three domains: medical treatment, care (e.g., routine care provided by nurses and care workers), and recuperation (e.g., how to spend their remaining life). By contrast, staff were asked about the challenges faced when promoting ACP.

### Participants and Recruitment Methods

We approached 14 Hansen’s disease sanatoria in Japan (13 national and 1 private) and invited individuals who had recovered from Hansen’s disease and resided in the sanatoria, together with the staff employed at these facilities, to participate in the study. Recruitment involved making a survey request to the sanatoria directors, the nursing directors, and the chairpersons of the self-governing councils, and inviting them to refer participants. On average, five ex-Hansen’s disease patients, one doctor, two to three nurses, two to three care workers, and one social worker participated per facility. We prioritized staff members who were engaged in daily care. Interviews were conducted from September 2015 to January 2017.

### Ethical Considerations

The study was conducted after obtaining approval from the Institutional Review Board (IRB) of St. Luke’s International University (No. 15-031). In addition, approvals have been obtained from the IRB of each facility, if necessary. All participants received a written explanation of the study’s purpose, methods, and confidentiality requirements, and we emphasized the voluntary nature of participation and their right to withdraw at any time. Regarding confidentiality, interviews were recorded, and notes were anonymized and stored securely in a locker to be kept on record for at least 5 years after publication in an academic journal. After that time, all records will be shredded and destroyed.

### Data Analysis

First, the interviews with staff and ex-Hansen’s disease patients were transcribed verbatim. Second, we carefully identified specific requirements of ACP for medical treatment, care, and recuperation from the interviews with ex-Hansen’s disease patients and summarized then in one sentence that aimed to maintain the original meaning, before assigning each sentence a code. Third, this process was repeated for responses from staff members, focusing on the challenges faced when implementing ACP in a sanatorium. Fourth, similar codes were classified into categories and subcategories per subject group. And we listed the narratives that represented each subcategory. All analyses were conducted in collaboration with the co-researchers to ensure validity. In the text, categories are enclosed in double quotation marks (i.e., “category”), whereas each subcategory is enclosed in single quotation marks (i.e., ‘subcategory’).

## Results

### Participant Characteristics

In total, 57 ex-Hansen’s disease patients (42 men, 15 women; mean age 82.4 years) from 10 facilities participated in the study. Among these, five of them (coded 6, 25, 18, 2, and 1) entered the sanatoria in the 1930s, 1940s, 1950s, 1960s, and 1970s, respectively. We did not know when another five individuals entered the sanatoria. There were 66 staff, comprising 27 nurses, 23 care workers, 10 physicians, and 6 social workers. The physicians included 8 directors and 2 vice-directors; the nurses included 3 nursing directors, 2 head nurses, 6 assistant head nurses, and 16 nurses; and the care workers included 13 head care workers, 2 sub-head care workers, and 8 care workers. The staff had a mean of 17.9 years of experience. The numbers attached after each narrative of the subjects indicate ex-Hansen’s disease patients (001–057) and the staff (058–123).

### Insecurities Related to ACP Among Ex-Hansen’s Disease Patients

When interviewing about the wishes for ACP among people who had recovered from Hansen disease, the following insecurities were uncovered and grouped into seven categories: “lack of physicians,” “insecurity regarding the quality of care provided by nurses and care staff,” “disintegration of the guardianship system,” “complicated family relationships,” “pressing need to confirm wishes and lack of information about AD initiatives,” “sanatorium-led property management,” and “future plans for continuance of the sanatorium.” For instance, the category on a anxiety about qualification as a nursing or caregiving staff and pressing need to confirm wishes and lack of information about AD initiatives and has been identified based on this statement made by an ex-Hansen’ disease patient:

Because they are working in a small group, it is important to raise the quality of each of them. But we found it difficult. As experienced staff are going out, new workers, who are not familiar with Hansen’s disease, are coming in. They cannot so much as make a judgment of the necessity of first aid. (045)

To illustrate, the category of Lack of information about AD initiative and accelerated patient’s confirmation of intention I do not think about my death is premised on statements such as this:

I was urged to confirm their intention of medical treatment they wished. A main topic of our conversation was life-sustaining treatments. (029)

**Fig. 1:**
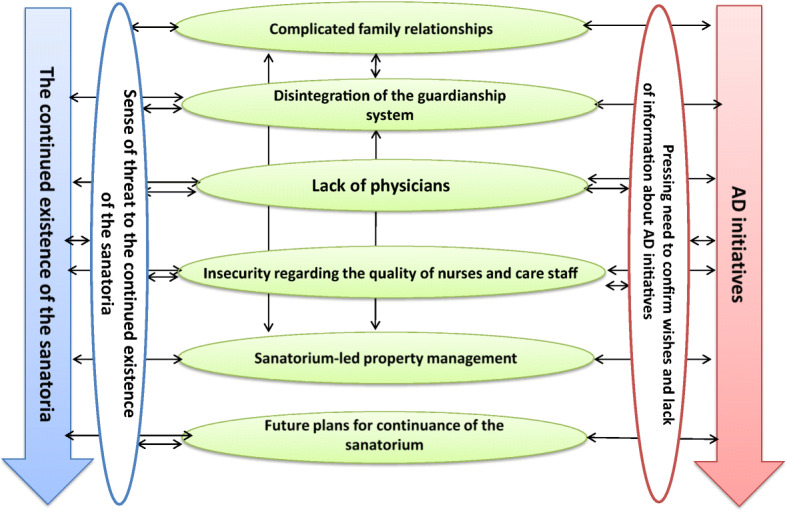
Insecurities related to ACP among ex-Hansen’ disease

Further examination of the relationships between categories produced the schema shown in Fig. 1. The diagram illustrated the correlation between the sanatoria’s AD/ACP initiatives that were taken in line with the government’s announcement about the retention of the sanatoria, and ex-Hansen’s disease patients’ anxieties and concerns about the initiatives. Insecurities persisted despite the enshrinement of rights in the 2008 Act to Accelerate the Resolution of Hansen’s Disease Problems. Because of the history of compulsory segregation, many survivors of Hansen’s disease had “complicated family relationships,” and because those entrusted with care in mutual help were aging, there was a concurrent “disintegration of the guardianship system.” Furthermore, the apparent “lack of physicians” to work in the sanatoria was viewed as a serious problem, as was “insecurity regarding the quality of care provided by nurses and care staff.” Thus, many survivors reported a “sense of threat to the continued existence of the sanatoria.”

Given that medical care in sanatoria is in a critical state, partly because ex-Hansen’s disease patients are increasing in age, sanatoria are now introducing AD. Many people living in a sanatorium, however, reported feeling confused by being “rushed into confirming their wishes” about terminal care and by having a “lack of information about AD initiatives.” Furthermore, after the lawsuit seeking damages by the government, sanatoria were worried about the increase in dementia associated with aging and had begun to introduce “sanatorium-led property management” involving contractors, which added to the issues of “complicated family relationships” and “disintegration of the guardianship system.” “Future plans for the continuance of sanatoria,” as part of the efforts for continuation of the sanatorium, were also beginning to be implemented.

### Staff opinions on the reasons to prevent promotion of ACP

As mentioned earlier, staff were aware of the need for ACP, yet it was not being fully implemented. Several reasons are given to explain this, as shown in Table 1. Table 1 indicates why ACP was not accepted in sanatoria from the sanatoria staff’s viewpoints. There were four core categories: ex-Hansen’s disease patients’ awareness of ACP; common awareness of ACP among ex-Hansen’s disease patients and staff in the sanatoria; care and approaches carried out by staff; administrative system and management. Below are the descriptions of the four core categories and narratives representing them.

**Table 1:** Staff opinions on the reasons not to promote ACP

Core category	Category	Subcategory
Awareness of ACP among ex-Hansen disease patients	Lack of sense of reality of end-of-life	I do not think about my death
		I am uninterested because I am healthy
		I wish to commit my end-of-life to my doctor
		I do not recognize the importance of advance directives on end-of-life care
	Insufficient understanding of life-sustaining treatments	Ex-Hansen disease patients were unable to understand technical words used in the explanation
		The explanation by staff was inadequate
		Ex-Hansen disease patients were unable to understand due to old age or hearing difficulties
	A sense of reserve toward the physician	The only choice is to spend my end-of-life with the doctor at the sanatorium
		I do not appeal directly to the doctor
	Valuing the wishes of patients’ distant family	Ex-Hansen disease patients have a feeling remorse makes them dependent on their family
Common awareness of ACP among ex-Hansen disease patients and staff in the sanatoria	Death (was) a taboo subject to be avoided	Ex-Hansen disease patients were annoyed at the terms, end of life and death
		Ex-Hansen disease patients feared death
		Ex-Hansen disease patients were in denial
		Staff discussed with the assumption of death
		It was an impenetrable topic
		A subject of death caused anxiety and negativity among ex-Hansen disease patients
	Place for dying and the practice of attending the deathbed	Staff thought that they should perform end-of-life care at the ward.
		Ex-Hansen disease patients thought that they should die and be given end-of-life care in the ward
		Ex-Hansen disease patients should avoid death in the living space out of courtesy for other residents
		Ex-Hansen disease patients should avoid troubles to other residents caused by end-of-life care in the living space
	Whether the relationship was trustworthy	Ex-Hansen disease patients refrained from saying real intention
		Ex-Hansen disease patients chose the staff with whom they would speak about ACP
		ACP could not be discussed without a relationship of trust
Care or approaches of staff	Staff-led approach centering on living will	Staff gave non-individualized explanations
		Staff favored the convenience of medical care providers
		Staff focused only on intention of end-of-life care
		Staff worked without considering ex-Hansen disease patients’ perspectives
		The number of patients who had completed their living wills was important
		After writing living will, there is no discussion at the end
	Insufficient team-based care	Staff were not aware of the significance of forming a team
		There was a lack of encouragement to promote coordination between nurses and care workers
		There was a lack of coordination between physicians
		There was a sparse communication or cooperation with other professionals and departments
		The delineations between the roles of nurses and care workers were excessive
		It was a physician’s role to listen to their wishes of life-sustaining treatments
	Insufficient information sharing between staff	Information obtained through years of individual relationships was not being utilized
		Lack of systems to share information was observed
		Staff were not recording information carefully
		Some care workers were unaccustomed to taking records
		Some care workers lacked computer skills
		They could not share a good practice of end-of-life care
	Physicians provided life-sustaining treatments based on their own values	Physicians gave life-sustaining treatments to terminally ill patients to feel secure
		Physicians worked without respecting patient’s wishes
	Insufficient knowledge and skills among medical caregivers	There were many physicians who do not have specialized knowledge to care for patients with Hansen disease
		There were some nurses who were unable to treat wounds caused by Hansen disease properly
		Physicians had insufficient knowledge or skills of end-of-life care for the elderly patients
		Nurses had a lack of knowledge, techniques, and observation abilities to care for ex-Hansen disease patients
		Nurses who did not differ from the perspectives of care workers had the same perspectives as care workers
	Medical caregivers were reticent to play an active role	Medical caregivers had low motivation to learn
		There were some physicians who did not get enough challenge in sanatoria
		There were some nurses who only performed routine tasks
		Physicians lacked awareness of their roles in AD
		None of the sanatoria provided home care service by physicians
	Anxieties about end-of-life care in residential settings	Insufficient night-shift system was observed
		There was a fear of care workers witnessing the death of residents
		Lack of education of care workers to provide end-of-life care was observed
	Fading awareness of the historical experiences of people with Hansen disease	History of Hansen’ disease was not passed onto new staff
		Some staff believed that ex-Hansen disease patients were luckier than the general elderly population
Organizational system and management	Weakening self-governance	Functions of mutual help are weakened as a result of old age of self-governing board members
		The wishes of the self-governing council do not necessarily represent those of all residents in the sanatoria
	Failure to understand the necessary roles of care workers in daily life	Staff showed little understanding of these necessary roles
		Staff were recognizing them as care workers under the nurses
		Care worker’s participation in the meetings is not promoted
		Some staff lowered the motivation of care workers
	Shortage of physicians	Lack of Hansen disease specialists was observed
		Lack of general physicians was observed
	Insufficient leadership for ACP	There were unclear approaches for ACP in sanatoria
		There was a lack of materials and manuals for communication
		There was a lack of strategies to communicate this approach
		There were no discussions to build a concrete system
	General trends behind the lag in implementation of new initiatives	There was a conservative idea in many facilities
		Staff accounted nursing directors function as dispatched managers
	Geographic or systemic issues preventing residents from dying in their own residences	Staff needed to walk long distances
		It was troublesome to cross mountains and valleys
		Infrastructure shortcomings were observed

#### Awareness of ACP Among Ex-Hansen’s Disease Patients

We derived four categories and ten subcategories regarding the awareness of ACP among ex-Hansen’s disease patients living in a sanatorium. Ex-Hansen’s disease patients had a “lack of sense of reality of end-of-life,” and this category is based on the following four subcategories: “I do not think about my death,” “I am uninterested because I am healthy,” “I wish to commit my end-of-life to my doctor,” and “I don’t recognize the importance of advance directives on end-of-life care.” To illustrate, the subcategory of “I do not think about my death” is premised on statements such as this:

Some residents in their 90s told that now was not a time to think about death. (115 nurse)

The category of “insufficient understanding of life-sustaining treatments” is based on the following three subcategories: ‘inability to understand technical words used in the explanation,’ ‘the explanation by staff was inadequate,’ and ‘unable to understand due to old age or hearing difficulties.’ To illustrate, the subcategory of ‘inability to understand technical words used in the explanation’ is premised on statements such as this:

Phonetic expression, kana, helped me read through the consent form, but it was difficult to understand the confusing words used there. (080, care worker)

The category of “a sense of reserve toward the physician” is based on the following two subcategories: ‘the only choice is to spend my end-of-life with the doctor at the sanatorium’ and ‘I do not appeal directly to the doctor.’ While the category of “valuing the wishes of patient’s distant family” is based on the following subcategory of ‘a feeling of remorse makes them dependent on their family.’

#### Common Awareness of ACP Among ex-Hansen’s Disease Patients and Staff in the Sanatoria

Three categories (“Death [was] a taboo subject to be avoided”, “place for dying and the practice of attending the deathbed”, “whether the relationship was trustworthy”) and 13 subcategories about ACP were derived from common responses given by ex-Hansen’s disease patients and the staff who treated them. Both groups felt that “Death [was] a taboo subject to be avoided” and this category is based on six subcategories: ‘Ex-Hansen’s disease patients were annoyed at the terms, end of life and death,’ ‘feared death’ and ‘were in denial,’ while staff perceived ACP as something ‘discussed with the assumption of death’ that was an ‘impenetrable topic’ that would ‘cause anxiety and negativity among ex-Hansen’s disease patients.’ This statement reveals that ex-Hansen’s disease patients were annoyed at the references to end-of-life and death:

A few people suddenly said, ‘My whole life has been messed up. I don’t want my terminal moment to be ruined any further.’ (118, doctor)

Another common thought concerned the preferred “place for dying and the practice of attending the deathbed,” and this category is based on four subcategories. The preference of the staff was that ‘they should perform end-of-life care at the ward.’ Ex-Hansen’s disease patients preferred ‘patients should die and be given end-of-life care in the ward’ and should ‘avoid death in the living space out of courtesy for other residents,’ thereby ‘avoiding troubles to other residents caused by end-of-life care in the living space.’ For instance, one participant said that patients should be given end-of-life care and die in the ward:

Ex-Hansen’s disease patients have rules in common. It is a long tradition to go to the ward at the time they need to be put on a drip. (094, nurse)

Finally, the category of “whether the relationship was trustworthy” extracted three subcategories. Both staff and ex-Hansen’s disease patients believed in the importance of “whether the relationship was trustworthy,” with patients commenting that they preferred to ‘refrain from saying their real intention,’ ‘choose the staff with whom they would speak about ACP,’ and staff commenting that ‘ACP could not be discussed without a relationship of trust.’ To illustrate, the subcategory of ‘ACP could not be discussed without a relationship of trust’ is premised on statements such as this:

I have confidence in listening to it. But I’ll do it very carefully on a basis of trust between us. (087, nurse)

#### Care or Approaches of Staff

The following 8 categories and 34 subcategories were extracted concerning how issues with staff care and approaches were barriers to promote ACP.

##### Staff-Led Approach Centering on Living Will

‘Non-individualized explanations’ were given that ‘favored the convenience of medical care providers,’ which ‘focused only on intention of end-of life care,’ ‘without considering ex-Hansen’s disease patients’ perspectives,’ level of preparation, or appropriateness of timing for the patient. Outcome was measured by ‘the number of patients who had completed their living wills,’ and after writing living will, ‘there is no discussion at the end.’ As this participant observed, the Staff favored the convenience of medical care providers:

We ask them, but we too much rely on their responses. We ask them basically because we don’t want to be confused. (059, nurse)

To illustrate, the subcategory of ‘the number of patients who had completed their living wills was important’ is premised on statements such as this:

Writing the (living will) shouldn’t be the only aim. It should be done very carefully. (078, doctor)

##### Insufficient Team-Based Care

There was a fundamental issue that ‘staff were not aware of the significance of forming a team.’ They reported that intra- and inter-professional coordination was lacking, such as ‘lack of encouragement to promote coordination between nurses and care workers’ and ‘lack of coordination between physicians,’ and ‘sparse communication or cooperation with other professionals and departments.’ ‘The delineations between the roles of nurses and care workers were excessive.’ In addition, the staff considered it to be the ‘a physician’s role to listen to their wishes of life-sustaining treatments.’ On the lack of encouragement to promote coordination between nurses and care workers, this participant observed:

I worked in a cooperative way only with staff of the same department. I thought that we should aggressively try to communicate and share information with team members such as nurses and care workers. (122, social worker)

##### Insufficient Information Sharing Among Staff

Despite working in a sanatorium for many years, it was noted by the participants that ‘information obtained through years of individual relationships was not being utilized.’ Explanations for this included ‘lack of systems to share information’ and ‘not recording information carefully.’ On the lack of systems to share information, a care worker observed:

I can’t handle too many sheets of paper that are not properly filed or shared among staff. I must spend a lot of work time on it. (119, care worker)

Some care workers were also ‘unaccustomed to taking records’ or ‘lacked computer skills.’ Although some people died in their rooms in line with their wishes, respondents commented that, for most people, ‘they could not share a good practice of end-of-life care.’

##### Physicians Provided Life-Sustaining Treatments Based on Their Own Values

Some physicians ‘gave life-sustaining treatments to terminally ill patients to feel secure.’ On this point, a doctor noted:

Life-sustaining treatments have been performed for a long time for doctors, not for someone else, to secure themselves. (078, doctor)

In some cases, physicians continued life-sustaining treatments ‘without respecting patient’s wishes.’

##### Insufficient Knowledge and Skills Among Medical Caregivers

For ex-Hansen’s disease patients who are suffering from after effects of the disease, care was given at the sanatoria by ‘physicians who do not have specialized knowledge to care for patients with Hansen’s disease’ and by ‘nurses who were unable to treat wounds caused by Hansen’s disease properly.’ To illustrate, the subcategory of ‘physicians who do not have specialized knowledge to care for patients with Hansen’s disease’ is premised on statements such as this:

Doctors here do not even know how to heal the wounds of ex-Hansen disease patients. (101, nurse)

It was thought that ‘physicians had insufficient knowledge or skills of end-of-life care for the elderly patients,’ while nurses had a ‘lack of knowledge, techniques, and observation abilities to care for ex-Hansen’s disease patients,’ such that ‘nurses who didn’t differ from the perspectives of care workers had the same perspectives as care workers.’ For instance, a nurse made this remark to illustrate a ‘lack of knowledge, techniques, and observation abilities to care for ex-Hansen’s disease patients’:

Nursing professionals work not just to confirm whether patients are well or not. I can’t understand the intention of some nurses who don’t measure patients’ blood pressure, they are holding a blood pressure gauge, though. (065, nurse)

##### Medical Caregivers Were Reticent to Play an Active Role

Both physicians and nurses had ‘low motivation to learn.’ The fact that ACP was not promoted was symptomatic of ‘physicians who didn’t get enough challenge in sanatoria’ and ‘nurses who only performed routine tasks.’ Staff other than physicians viewed creating AD as the physician’s role, but the physicians themselves ‘lacked awareness of their roles in AD.’ Medical care in a sanatorium was mainly given on an outpatient basis, except when in medical wards. Although many ex-Hansen’s disease patients wanted home care service by physicians because of their advanced age, ‘none of the sanatoria provided this service.’ In this respect, a participant observed:

Some doctors, without hesitation, make patients with a fever come to see them as an outpatient. Some other doctors occasionally visit patients to give a medical consultation, not to see their daily life in sanatoria. (093, nurse)

##### Anxieties About End-of-Life Care in Residential Settings

In sanatoria, dying patients are traditionally moved from residences to the medical ward. It was recognized that some individuals prefer to die in their own residence, but staff were anxious about this because of an ‘insufficient night-shift system,’ ‘fear of care workers witnessing the death of residents,’ and ‘lack of education of care workers to provide end-of-life care.’ A participant noted:

I have not received specific education, I have not attended a conference, I really feared how I should respond when patients die in front of my eyes. (112, care worker)

##### Fading Awareness of the Historical Experiences of People with Hansen’s Disease

Although staff believed it was important to provide care with full understanding of the histories of ex-Hansen’s disease patients, the ‘history of Hansen’s disease was not passed onto new staff,’ such that some staff believed that ‘ex-Hansen’s disease patients were luckier than the general elderly population’ because they received a public pension or compensation. The participants pointed out that such misunderstanding was an important problem. The failure to properly prepare new staff was noted by a participant:

It is our mission to recognize that residents here are those who have challenged the issues of Hansen’s disease. We should remind ourselves of the mission and our expertise gained by nursing experience here. (073, nurse)

#### Organizational System and Management

The following 6 categories and 17 subcategories are concerned with how issues with the organizational system and management were obstacles to promoting ACP.

##### Weakening Self-governance

At first, the ‘functions of mutual help are weakened as a result of old age of self-governing board members.’ Most board members on the self-governing council remain relatively healthy, but there is a wide spread of health states among residents, and thus many staff felt that ‘the wishes of the self-governing council do not necessarily represent those of all residents in the sanatoria.’ This participant noted:

They cannot go to the next room without someone’s help and adequate care. Many of them don’t want to trouble their staff even though they like to visit a room of their friends. They are isolated. (096, nurse)

##### Failure to Understand the Necessary Roles of Care Workers in Daily Life

Care workers help with cleaning living spaces, preparing meals, and assisting with dressing for patients who are completely blind or have multiple disabilities. They have a close and important role in supporting residents, but medical staff were reported to ‘show little understanding of these necessary roles,’ only recognizing them as ‘care workers under the nurses.’ Therefore, in sanatoria, it was often believed that care workers did not need to attend care meetings if head nurses attended, thereby ‘Care worker’s participation in meetings was not prompted,’ although they have useful care-related information obtained through close interactions with residents. This also ‘lowered the motivation of care workers’ to promote ACP. A participant explained how a staff showed little understanding of these necessary roles:

Care workers provide much care except what is written on papers. Superiors of the care workers cannot be informed of unexpressed care. (096, nurse)

##### Shortage of Physicians

Although dermatologists, ophthalmologists, and otolaryngologists often see Hansen’s disease outpatients due to the symptoms, general physicians as well are needed to examine the whole condition of elderly residents. However, in addition to the ‘lack of Hansen’s disease specialists,’ the sanatoria in this study were deemed to have a ‘lack of general physicians.’ A participant explained lack of general physicians:

Ex-Hansen disease patients are most concerned that there are no general physicians in the sanatoria now. (088, nurse)

##### Insufficient Leadership for ACP

Participants explained that leadership was needed to promote ACP. They thought there were ‘unclear approaches for ACP in sanatoria,’ with a ‘lack of materials and manuals for communication,’ a ‘lack of strategies to communicate this approach,’ and ‘no discussions to build a concrete system.’ This participant highlighted the need to build a concrete system:

The scope of my support to ex-Hansen’s disease patients has its limits. I hope that an authorized framework to start with will be created. (094, nurse)

##### General Trends Behind the Lag in Implementation of New Initiatives

Staff said there was a ‘conservative idea in many facilities.’ It is usually the case that nursing directors transfer to other national facilities every third year according to the custom of the national organization. Because of these circumstances, staff recognized that ‘nursing directors function as dispatched managers,’ which may explain why there is a lag in implementation of ACP. A participant explained this conservatism as follows:

Staff are unwilling to do something for their patients. They do not want any change to happen to themselves. They do not want to be troubled. Doing things on a daily basis for patients is not annoying, but they want to avoid doing new things. (110, doctor)

##### Geographic or Systemic Issues Preventing Residents from Dying in Their Own Residences

The legacy of compulsory segregation remains in the geographic locations of some sanatoria that are in distant and isolated places. If ex-Hansen’s disease patients are to die in their own rooms, medical care providers would be required to travel ‘long distances’ and ‘cross mountains and valleys’ to reach them. Another issue was ‘infrastructure shortcomings,’ such as the lack of medical equipment in residences. To illustrate, the subcategory of ‘infrastructure shortcomings’ is premised on statements such as this:

There is a physical limit to the residences of ex-Hansen disease patients. Their rooms are not wide enough for stretchers and wheelchairs. (060, nurse)

## Discussion

### The Need for a Perception Shift from AD to ACP

AD were actively organized at the target facilities, but staff acknowledged that efforts only focused on completing the AD without considering the state of preparedness and individuality of ex-Hansen’s disease patient. Such problems have been observed in prior research despite differences in facilities (Weathers et al. 2016). Somewhat similar to the staff, ex-Hansen’s disease patients were uneasy about confirming their wishes for terminal care and the AD, which they felt was being rushed without adequately understanding the need for AD or their wishes for the future. Choices for end-of-life care are private and related to a person’s lifestyle and way of life. Staff-led efforts to promote AD could be used as weak enforcement, considering the unique circumstances of ex-Hansen’s disease patients who have been discriminated against and have been isolated from their families and society.

An issue underlying the efforts to promote AD is that their documentation can easily become a concrete numerical target of compliance in sanatoria. This is supported by the fact that AD have been used as an outcome measure for completing ACP in prior research (Brown et al. 1999; Molloy et al. 2000; Morrison et al. 2005). As stated above, a notice issued by the Ministry of Health, Labor and Welfare has also mentioned the need for efforts to understand end-of-life care wishes (the Ministry of Health, Labor and Welfare, 2014). Information gathered from both staff and ex-Hansen’s disease patients indicated that a perception change is needed to understand that AD are part of the ACP process and not the goal. Polite daily care and communication nurtured from daily care may be key, and the way that ACP is initiated is important. Indeed, ACP should be viewed as a process in which AD-related issues should not be asked from the outset (Norals and Smith 2015). When wishes are conveyed by ex-Hansen’s disease patients, if they can be discussed and realized, we might increase quality of life, protect dignity, and ultimately promote ACP.

### Promoting Communication of ACP Through Daily Care

The previous studies (Aultman et al. 2018; Dixon and Knapp 2018; Fulmer et al. 2018) indicated that lack of time for healthcare providers to spend for ACP appeared to be a barrier to its promotion. However, in Hansen’s disease sanatoria in Japan, hospital and residential areas are in the same place; therefore, it is easier to discuss the wishes and hopes of ex-Hansen’s disease patients. Additionally, residents have often lived in sanatoria for prolonged durations, so these become their homes during the final stages of life. Given that sanatoria are also places of residence, there are several features regarding daily contact that provide opportunities to talk directly to ex-Hansen’s disease patients about their wishes, such as regular home care visits by nurses, diagnoses by doctors, and everyday life support by care workers, rather than simply going through the standard interactions or safety checks. To promote ACP, it is important to have relationships that make it possible to talk openly, which might be facilitated by having continued contact and creating a favorable environment (Tsuruwaka et al. 2016). However, problems with staff care were a perceived reason for difficulties in promoting ACP. The main issues related to team-based care and information sharing, which were considered inadequate. For a team to function, it is important that each member is respected and that his or her role is understood, including the focus of each in the multidisciplinary setting (Golom and Schreck 2018). Regular meetings could facilitate this to promote cooperation among staff, while making the vision of the institution clear.

Even though staff listened to the modest hopes expressed by ex-Hansen’s disease patients, we found that there was no effective way for staff to share this information, creating a situation that precluded specific support being offered. Information sharing could be improved by regular meetings and having a centralized way of recording information received from ex-Hansen’s disease patients. Efforts are also needed to determine how best to record communication among staff, and indeed, what should be recorded and whether it would help with ACP.

Nurses most often implement communication about ACP (Hinderer and Lee 2014); however, in sanatoria, just like facility for the elderly, there are many care workers. Therefore, communication through daily care between the care workers and ex-Hansen’s disease patients can play an important role to promote ACP. Nevertheless, the role of the care workers, which is the heart of supporting daily activities, has been poorly understood and has not been used to drive the fostering of ACP. Managers need to understand that care workers can provide invaluable assistance with promoting ACP. Creating environments that allow caregivers to participate actively in meetings would facilitate this role.

We are also conducting a survey about nursing care in Hansen’s disease’s sanatoria under Japan’s control in Taiwan (Tsuruwaka and Yokose 2014) and South Korea. When considering the condition of elderly ex-Hansen’s disease patients living in sanatoria, it is clear that, as in Japan, there is an urgent need to promote ACP with focus on the individual. There are limits in this study as follows. First, Japan’s exceptional segregation policies that excluded Hansen’s disease patients for a long period make it difficult to generalize their current situations. Second, most participants in the current study who were in consideration for sanatoria, were in relatively good health, and could still communicate; and those who lived independently in rooms. In thinking about ACP, more information is required for people of different age groups and with different health conditions.

### Conclusion

We believe that there is a need to implement the wishes ex-Hansen’s disease patients if we are to generate meaningful ACP with focus on the individual. Given that the patients live in a sanatorium, listening to their wishes can be nurtured through meticulous team-based care. ACP implementation urgently requires better organization building effective interdepartmental cooperation and an enabling framework, among other conditions that this study has identified.
